# Gradient Functionalization of Poly(lactic acid)-Based Materials with Polylysine for Spatially Controlled Cell Adhesion

**DOI:** 10.3390/polym16202888

**Published:** 2024-10-14

**Authors:** Viktor Korzhikov-Vlakh, Aleksandra Mikhailova, Ekaterina Sinitsyna, Evgenia Korzhikova-Vlakh, Tatiana Tennikova

**Affiliations:** 1 Institute of Chemistry, St. Petersburg, St. Petersburg State University, 198504 St. Petersburg, Russia; a.mikhailova@vir.nw.ru (A.M.); kat_sinitsyna@mail.ru (E.S.); t.tennikova@spbu.ru (T.T.); 2 St. Petersburg State University Hospital, 199034 St. Petersburg, Russia; 3 Federal Research Center N. I. Vavilov All-Russian Institute of Plant Genetic Resources (VIR), 190000 St. Petersburg, Russia

**Keywords:** poly(lactic acid), surface gradient modification, polylysine covalent grafting, thiol-ene click reaction, biomedical materials

## Abstract

The development of biomaterials with gradient surface modification capable of spatially controlled cell adhesion and migration is of great importance for tissue engineering and regeneration. In this study, we proposed a method for the covalent modification of PLA-based materials with a cationic polypeptide (polylysine, PLys) via a thiol-ene click reaction carried out under a light gradient. With this aim, PLA-based films were fabricated and modified with 2–aminoethyl methacrylate (AEMA) as a double bond source. The latter was introduced by reacting pre-formed and activated surface carboxyl groups with the amino group of AEMA. The success of the modification was confirmed by ^1^H NMR, Raman and X-ray photoelectron spectroscopy data. A further photoinduced thiol-ene click reaction in the presence of a photosensitive initiator as a radical source was further optimized using cysteine. For grafting of PLys via the thiol-ene click reaction, PLys with a terminal thiol group was synthesized by ring-opening polymerization using Cys(Acm) as an amine initiator. Deprotection of the polypeptide resulted in the formation of free thiol groups of Cys-PLys. Successful gradient grafting of Cys-PLys was evidenced by covalent staining with the fluorescent dye Cy3-NHS. In addition, PLys gradient-dependent adhesion and migration of HEK 293 cells on PLys-PLA-based surfaces was confirmed.

## 1. Introduction

Tissue engineering is an actively growing interdisciplinary field focused on the development of materials for regeneration of damaged tissues using adhered primary cells [1,2]. There are several aspects that need to be considered when developing materials for tissue engineering. First, such materials should have mechanical characteristics typical of the target tissues and should be able to support three-dimensional (3D) cell growth [3,4]. Second, they should contain on their surface factors (signaling molecules) that improve cell adhesion and cell growth and ensure the organization of cells into a new functional tissue [5,6].

Biodegradable polymers based on lactic acid occupy an important place in designing scaffolds [7,8]. The reasons for this interest are biocompatibility and the biodegradability of poly(lactic acid) (PLA), whose biodegradation products can be utilized in vivo by natural metabolic pathways (e.g., Cori cycle) [9,10,11]. The application of PLA and its copolymers in the development of biomaterials is that this polymer is approved by the American Agency for Food and Drug Administration (FDA). In particular, PLA is now widely used for the preparation of suture threads, various 3D scaffolds for tissue regeneration, and micro- and nanoparticles for drug delivery [12,13].

However, being a promising polymer for biomedical applications, PLA reveals a number of disadvantages associated with the chemical structure of the polymer chain. To date, methods of modification of both the polymer in the volume and on the surface of PLA-based materials are being actively developed. The main disadvantages of this polymer are its relatively high hydrophobicity (the contact angle of wetting with water is more than 80°) [14] and the lack of reactive groups on the surface. This may lead to uncontrolled nonspecific adsorption of proteins on the surface and the impossibility of direct attachment of bioactive molecules to the surface of PLA-based materials. In addition, neat PLA-based materials exhibit their own poor cell adhesion and proliferation [15]. The latter can be enhanced by additional surface hydrophilization or functionalization with positively charged polypeptides [16,17,18], growth factors [19,20], RGD peptides [21,22], and other bioactive molecules [23].

Often, PLA materials are modified with natural polymers in order to increase the degree of cell adhesion on the surface. The most commonly used strategies for pre-modification of PLA surfaces include plasma treatment or partial-surface PLA hydrolysis [24,25]. For example, O_2_ plasma treatment was reported to increase the wettability and adhesion of nerve cells on the surface of PLA films [26]. Alkaline hydrolysis is the simplest way to create reactive carboxyl and hydroxyl groups, which can then interact with modifying molecules containing amine (−NH_2_) and hydroxyl (−OH) functionalities. For instance, using the preliminary alkaline hydrolysis followed by activation of carboxylic groups generated on the PLA surface, it was possible to bind chitosan covalently [27]. As a result of such modification, the attachment and proliferation of rat osteoblasts on the surface of polymer materials significantly improved. A similar approach was developed in our group for covalent modification of the surfaces of PLA- and PCL-based materials of different designs with synthetic hydrophilic glycopolymer capable of functionalization with bioligands [28]. In that case, PLA/PCL surfaces were partly hydrolyzed with sodium hydroxide, formed carboxylic groups were transferred to activated esters and treated with ethylenediamine to get an amino-bearing surface capable of a reaction with aldehyde-bearing glycopolymer. Using a similar approach, the modification of the PLA surface with a covalently bound hydrophilic layer of polyacrylamide or alginate hydrogel was successfully performed [29]. Pellegrino et al. proposed a method of covalent grafting of taurine by aminolysis of the PLA surface to increase its hydrophilicity [30]. Taurine is a natural, non-toxic compound and has a nucleophilic primary amine and a sulfo group. After taurine grafting, the surface acquired a strong negative charge due to sulfo groups of grafted taurine and was further used for collagen adsorption due to electrostatic interactions. In vitro experiments demonstrated the ability of the modified PLA materials to support chondrocyte adhesion and proliferation on the surface compared to the unmodified PLA surface. Intermediate ammonolysis of PLA scaffolds was also used by Haddad et al. for further covalent modification of the surface with epidermal growth factor (EGF) [19]. Other advances in the non-covalent and covalent modification of PLA-based materials are summarized in some recent reviews [15,31,32].

Besides the development of methods to modify the surface of PLA-based materials, there is a need to create scaffolds with a spatially controlled arrangement of bioactive factors that determine the formation of interfaces between different tissues, e.g., bone and other tissues, including cartilage tissue, ligaments, and tendons. In natural conditions, these surfaces are not separate zones with sharp transitions in properties but possess certain physical and biochemical gradients [21,33]. The composition of biochemical factors and the organization of cell types in the extracellular matrix change gradually during the transition between different tissues. The development of methods for gradient surface modification of materials intended to be used as scaffolds in tissue-engineered tissues is of great importance. For instance, a gradient of adhesion factors can promote better cell penetration inside the scaffold [21,34].

Over the past decade, various techniques have been developed to create gradients of density, stiffness, porosity, and surface functionality in biomaterials [34,35,36,37,38]. As to surface functionalization, polymer surfaces are usually modified with biologically active macromolecules that promote cell proliferation and migration [39,40]. Using a developed technique based on the application of fluorescent tags, it has become possible to visualize cell migration in vivo [41]. For instance, Brandley et al. showed that cells are noticeably distributed on the polyacrylamide gel surface with a gradient of RGD–peptide [42]. Moreover, the cell density was significantly higher in the region of the gel where the concentration of the peptide was higher. Wu et al. showed that a surface with a gradient density of heparin allows further creation of a gradient of fibroblast growth factor [43,44]. This, in turn, led to the selective adhesion of vascular smooth muscle cells with 70% of their directed migration. Vega et al. developed a thiol–norbornene-modified hyaluronic acid hydrogel functionalized by spatially varied light exposure with gradients (0–5 mM) of peptides that mimic cell–cell or cell–matrix interactions [45]. Similarly, Fisher et al. proposed a hyaluronic acid hydrogel crosslinked with matrix metalloproteinase cleavable peptides and modified with multiphoton labile nitrodibenzofuran for gradient photochemical immobilization of epidermal growth factor [46]. Gradient photoinitiated grafting of semaphorin 3A (neuronal membrane protein) into a Matrigel-based scaffold for cortical tissue regeneration was reported by Xu et al. [47].

Although gradient modification has been widely discussed for various types of materials, only a few studies have focused on the gradient modification of aliphatic polyesters. For example, Zhang et al. reported the fabrication of films based on a copolymer of L–lactic acid and a furan–maleimide functionalized trimethylene carbonate-bearing covalently immobilized laminin-derived peptide [48]. The latter, containing terminal thiol functionality, was grafted using the thiol-ene click reaction onto the surface of a polyester film micropatterned by the polydimethylsiloxane mold-pressing method. Zhu et al. created a linear density gradient of alendronate on the surface of aminolyzed PCL membrane. The gradient of alendronate promoted the gradient osteogenic differentiation of bone mesenchymal stem cells [49].

**Figure 1:**
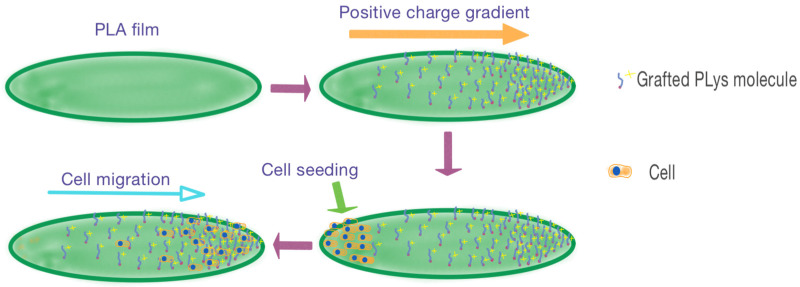
General idea of the study: formation of positive charge density gradient on the surface of the PLA-based films in order to induce cell migration.

In this study, we proposed a new approach to obtain gradient modification of PLA surface with poly–L–lysine (PLys) using thiol-ene click chemistry (Figure 1). PLA-based films were utilized as a convenient material for the development of a surface modification technique. The modification of the surface of PLA films was carried out in several steps and involved surface functionalization with linker-containing double bonds followed by their reaction with terminal thiol groups of PLys under concentration gradient conditions. The obtained materials were tested for adhesion of human embryonic kidney cells (HEK 293) and the ability of their migration within the surface.

## 2. Materials and Methods

### 2.1. Materials

D,L–lactide, tin octoate, *N*–(3–dimethylaminopropyl)–*N*–ethylcarbodiimide (≥98.0%), *N*–hydroxysuccinimide (≥99.7%), 2–(*N*–morpholino)ethanesulfonic acid (MES) (≥99.5%), 2–aminoethyl methacrylate (≥90.0%), diisopropylamine (≥99. 0%), ε–carbobenzyloxy–L–lysine (Lys(Z), ≥99.0%), L–cysteine (≥99.0%), bis(trichloromethyl)carbonate (≥98.0%), α–pinene (≥99%), mercury(II) acetate (≥98.0%), silver tetrafluoroborate (≥99.0%), anisole (≥99.7%), triphenylphosphine (≥99.0%), 5,5′- dinitrobis(2–nitrobenzoic acid) (≥98.0%) (DTNB), lithium phenyl–2,4,6–trimethylbenzylphosphinate (LAP, ≥95.0%), trifluoroacetic acid (TFA) (≥99.9%), trifluoromethanesulfonic acid (TFMSA), *S*–acetomidomethyl–L–cysteine hydrochloride (Cys(Acm), ≥99.0%) were purchased from Sigma–Aldrich (St. Louis, MO, USA) and used without prior purification except for D,L–lactide. Organic solvents, namely chloroform, toluene, methanol, diethyl ether, 1,4–dioxane, acetic acid, petroleum ether, etc., were purchased from Vecton (St. Petersburg, Russia). Prior to the synthesis of *N*–carboxyanhydride (NCA) of Lys(Z), dioxane, ethyl acetate, and petroleum ether were purified according to standard procedures. CDCl_3_ and DMSO-d6 were purchased from Astrachim (St. Petersburg, Russia). Sodium tetraborate (≥99.5%) was produced by Merck (Darmstadt, Germany), potassium and sodium chloride by Vecton (St. Petersburg, Russia), sodium hydrophosphate, potassium dihydrophosphate, and 2–(*N*–morpholino)ethanesulfonic acid (MES, ≥99.5%) by Sigma–Aldrich (St. Louis, MO, USA). The polymer solutions were purified before film casting using Millipore Millex–FG polytetrafluoroethylene (PTFE)-based syringe hydrophobic filters with a pore size of 0.45 μm (FilterBio, Nantong City, China).

### 2.2. Methods

#### 2.2.1. Synthesis of PLA

PLA was synthesized by ring-opening polymerization (ROP) of D,L–lactide. Prior to the polymerization, D,L–lactide was purified by recrystallization from toluene. For polymerization, 4.5 g of monomer was placed into a 15 mL Schlenk flask. Using a syringe, 2.0 mL of *n*–hexane was added through a sealing rubber septum. Next, the calculated amount of tin octoate (SnOct_2_) dissolved in a small amount of *n*–hexane was added to the monomer solution ([M]/[I] = 2500). The content of the flask was vacuumized with simultaneous intensive stirring and increasing the temperature to 130 °C using a glycerol bath. At the same time, *n*–hexane was removed from the reaction mixture and condensed in a freezing trap. Polymerization was carried out at 130 °C in the mass of the monomer for 4 h. At the end of the process, the polymerization system was cooled to room temperature. The solid mass was dissolved in chloroform. The resulting solution was concentrated using a rotary evaporator, and the polymer product was precipitated into cold methanol. The yield of PLA was found to be 75%. The structure was confirmed by ^1^H NMR spectroscopy (ppm, CDCI_3_): 1.56–1.62 (m, 3H, CH_3_), 5.15–5.23 (m, H, CH).

#### 2.2.2. Synthesis of Cys(Acm)-PLys(Z)

The polymerization was performed by ROP of Lys(Z) NCA. The latter was synthesized and purified prior to polymerization. Briefly, 2.1 g (7.49 mmol) of Lys(Z) and 150 mL of 1,4–dioxane, previously distilled over metallic sodium, were placed in a 250-mL three-neck flask equipped with a thermometer, a chlorocalcium tube, and an argon communication. Then, 0.74 g (2.5 mmol) of bis–(trichloromethyl)carbonate was introduced to the reaction flask. To bind the released hydrogen chloride, 4.6 mL of α–pinene was also added to the reaction mixture. The synthesis was performed at 56 °C. One hour after the start of the reaction, 0.037 g of bis–(trichloromethyl)carbonate was added to the reaction mixture. Additions were repeated twice per hour. The synthesis was carried out for 5 h until the reaction mixture became visibly clear. Finally, the solvent was evaporated, and anhydrous petroleum ether was added to the concentrated residue. The flask was left for 24 h at 4 °C until a white precipitate of Lys(Z) NCA appeared. The reaction product was purified by recrystallization from ethyl acetate. The purified product was dried in a vacuum exicator. The yield of the product was 90%. The structure was confirmed by ^1^H NMR spectroscopy (CDCI_3_, ppm): 1.34–1.65 (m, 4H, CH_2_CH_2_), 1.73–2.03 (m, 2H, CH_2_), 3.18–3.25 (m, 2H, NHCH_2_), 4.28 (t, 1H, CH), 4.92 (s, 1H, NH), 5.13 (s, 2H, C_6_H_5_CH_2_O), 6.73 (s, 1H, NH), 7.28–7.41 (m, 5H, C_6_H_5_).

The polymerization of Lys(Z) NCA was carried out using Cys(Acm) as the initiator. The reaction mixture was prepared in freshly purified and anhydrous DMF so that the monomer concentration was 4%. Before polymerization, a stock solution of initiator was prepared by dissolving Cys(Acm) in DMF. The ratio of monomer/initiator was set as 20. Polymerization was carried out for 72 h at 25 °C under shaking. The polymer products were purified by dialysis against water using a dialysis bag with MWCO 1000 for 48 h. Finally, the purified samples were freeze-dried. The yield of the polymer was 62%. The structure of Cys(Acm)-PLys(Z) was confirmed by ^1^H NMR spectroscopy (DMSO-d6, ppm): 1.15–1.64 (m, 3H, CH_3_), 1.96 (s, 3H, CH_3_CO), 2.96 (s, 2H, NHCH_2_), 4.06 (m, 2H, CH_2_S), 4.22 (s, 2H, 2CH), 4.37 (t, H, NHCH_2_S), 5.03 (s, 2H, CH_2_-C_6_H_5_), 7.18–7.37 (m, 5H, C_6_H_5_), 7.87–8.55 (s, 4H, 4 NH).

#### 2.2.3. Polypeptide Deprotection

##### Z-Group Removal

The Z-protecting group was removed by TFMSA/TFA solution. With this aim, 0.5 g of Cys(Acm)-PLys(Z) was dissolved in 20 mL of TFA, and the resulting solution was cooled under stirring in an ice bath. After 30 min, 1.0 mL TFMSA (5% regarding TFA) was added and the deprotection was carried out for 3 h at room temperature and constant stirring. After that, the polymer was precipitated in diethyl ether, isolated by centrifugation, and freeze-dried. The yield was 78%.

##### Acm-Group Removal

The removal of the Acm-protective group was performed using two approaches. The first one was based on the polymer treatment with mercury(II) acetate in acetic acid. In this case, 0.1 g of Cys(Acm)-PLys was dissolved in 10% acetic acid. After adjusting the pH of the solution with glacial acetic acid to the value of 4, 1 eq mercury acetate (43.1 mg) was added to the reaction mixture. The reaction proceeded for 1 h in an argon atmosphere under stirring at 22 °C. Then, 500 μL of *β*–mercaptoethanol was added to the reaction mixture, which was left under stirring for 5 h. The light-yellow colored powdery precipitate was separated by centrifugation, and the supernatant was purified by dialysis (MWCO 1000) against water. The deprotected product was freeze-dried. The product yield was 74%.

The second approach was based on the polymer treatment with AgBF_4_ in TFA. With this aim, 0.3 g of Cys(Acm)-PLys was dissolved in 100 mL of cooled TFA (4 °C). Then, 1.1 mL of anisole and 20 eq of AgBF_4_ per Acm-group were added to the polymer solution. The reaction mixture was incubated at 4 °C for 2 h. After that, cold diethyl ether was added to precipitate polypeptide. The precipitate was separated by centrifugation and dissolved in 1M acetic acid. Triphenylphosphine (20 eq per Acm group) was added to the resulting mixture and stirred at room temperature for 4 h. Finally, the supernatant containing the product was separated from the precipitate by centrifugation and concentrated on a rotary evaporator. The concentrate was purified by dialysis against water (MWCO 1000) for 24 h followed by freeze drying of the product. The product yield was 65%.

#### 2.2.4. Characterization of Polymers

The molecular weights of polymer products were determined by size-exclusion chromatography using an LC-10A Shimadzu (Kyoto, Japan) chromatographic system equipped with refractometric RID-10A detectors, controller SCL-10A, and two Agilent PLgel Mixed-D columns connected in tandem. LC Solution 1.24SP1 software was used for data processing. The analysis of PLA was carried out in THF at 40 °C. Molecular weights were calculated regarding the calibration curve built with the use of polystyrene standards (4000 ≤ M_w_ ≤ 498,000; Đ ≤ 1.29). The analysis of Cys(Acm)-PLys(Z) was carried out in DMF containing 0.1 M LiBr at 60 °C. Molecular weights were calculated regarding the calibration curve built with the use of poly(methyl methacrylate) standards (1000 ≤ M_w_ ≤ 250,000; Đ ≤ 1.40).

^1^H NMR spectra of monomers and polymers were recorded with the use of a Bruker Avance III (400 MHz) spectrometer in CDCl_3_ or DMSO-d6.

To determine the free SH groups in Cys-Plys, a reaction with Ellman’s reagent (5,5′–dinitro–bis–(2–nitrobenzoic acid), also known as DTNB) was carried out. The method is based on the binding of DTNB to thiol groups in solution. This reaction produces 2–nitro–5–thiobenzoic acid (TNB), which has a high extinction coefficient in the visible wavelength range (400–760 nm). For the analysis, 0.1 M sodium phosphate buffer containing 1 mM EDTA, pH 8, was used as a reaction medium. Ellman’s reagent solution was prepared as follows: 4 mg of dry reagent was dissolved in 1 mL of the reaction buffer. The calibration curve was built for the free cysteine dissolved in the reaction buffer. With this aim, 50 µL of Ellman’s reagent solution was added to 2.5 mL of the cysteine solution in the range of concentrations 0.25–1.50 mM. The solutions were mixed thoroughly and left for 15 min at room temperature. The absorbance of each solution was measured using a spectrophotometer at a wavelength of 412 nm. In the case of Cys-PLys, the procedure was the same as for cysteine solutions. The polymer concentration used for analysis was 0.5 mM. The amount of thiol groups in polymer samples was calculated with the use of a pre-built calibration curve for cysteine.

#### 2.2.5. Manufacturing of PLA-Based Films

PLA-based films were formed by casting the polymer solution in chloroform onto a cellophane substrate fixed on the glass ring. One film requires 0.1 g of PLA, which was dissolved in 1.2 mL of CHCl_3_. The resulting solution was poured onto the surface cellulose film fixed on a 30 mm diameter glass ring. After evaporation of the solvent, the films were formed on the cellophane surface. To facilitate manipulation during modification steps before the thiol-ene click rection, the films were not detached from the supporting construction. The thickness of the prepared films was measured using a micrometer.

#### 2.2.6. Covalent Modification of PLA Film Surface

##### Saponification and Activation of Carboxyl Groups

In order to increase the number of reactive carboxyl groups on the surface of PLA-based material, the surface of the PLA films was treated with 10 mL of 0.1 M NaOH solution for 30 min at room temperature. After that, the film was washed several times with distilled water until the neutral pH of the washing solution was achieved.

The generated surface carboxyl groups were activated using 0.1% DIC/NHS solutions in MES buffer, pH 5.4, cooled to 4 °C. For activation, 2 mL of DIC solution was added to the film and incubated for 10 min. After that, 2 mL of NHS solution was placed onto the surface of the PLA film. The reaction was carried out under vigorous shaking for 30 min. Finally, the film was washed several times with distilled water.

##### Surface Modification with AEMA

The commercially available AEMA HCI was converted to free amine under the action of diisopropylethylamine. With this aim, 450 μL of diisopropylethylamine was added under stirring to 0.3 g of AEMA HCI dissolved in 15 mL of diethyl ether. After 2 h, the formed precipitate was separated by centrifugation. The solvent from the supernatant obtained was removed by a rotary evaporator. In the next step, the PLA film surface was modified with AEMA. A solution containing 100 µL of AEMA in 3 mL of 0.025 M borate buffer (pH 8.5) was prepared and added to the surface of the film. The modification reaction was carried out for 8 h under constant shaking and at room temperature. The modification was testified by ^1^H NMR spectroscopy (DMSO-d6, ppm): 1.19–1.61 (m, 3H, CH_3_), 1.87 (s, 3H, CH_3_CO), 3.68 (s, 2H, CH_2_NH), 4.17–4.22 (m, 2H, CH_2_O), 5.15–5.22 (m, H, CH), 8.31 (s, 1H, NH).

##### Gradient Formation: thiol-ene Click Reaction

A 0.5 mL solution containing 5 mg/mL of Cys or Cys-PLys and 0.5 mg/mL of lithium phenyl–2,4,6–trimethylbenzylphosphinate (LAP, photoinitiator) in acetate buffer, pH 5.5, was added to the wells of a 6-well plate. A film was placed over the solution so that the AEMA-modified side of the PLA film was in contact with the solution. The maskless projection photolithography method was applied with Smart Print UV (Microlight 3D, La Tronche, France) to form the gradient on the film surface. For that, the plate was placed on the pad of the Smart Print UV so that the light flux was directed to the well with the sample film. The radical thiol-ene reaction between terminal -SH groups of Cys or Cys-PLys and AEMA on the surface of the PLA films was induced with the application of a 405 nm (25 W) LED source. The exposure time varied from 5 to 20 min. The grafting density gradient was provided by the gradient of light intensity, which in turn was achieved by the projection of a predesigned Adobe Photoshop (version 21) digital pattern (Figure S1, Supplementary Materials) onto the reflecting digital mirror device of a Smart Print UV. After the reaction, the film surface was washed three times with PBS.

##### Treatment of the Film Surface with Cy3-NHS Dye

For visual control of the grafted Cys or Cys-PLys, the surface of the film was treated with Cy3-NHS dye (λ_ex_ = 555 nm, λ_em_ = 570 nm). With this aim, 10 μL of a solution containing 1 mg/mL of Cy3-NHS in DMSO was added to 3 mL of 0.025 M borate buffer, pH 8.4. The resulting solution was placed on the surface of the gradient-modified film with Cys and Cys-PLys. After 2 h, the film surface was carefully washed with sodium phosphate buffer.

#### 2.2.7. Determination of Functional Groups on PLA Film Surface

##### Carboxyl Groups after Saponification and Activation

The number of carboxyl groups on the film surface after saponification and activation was determined by the color reaction of glycine with ninhydrin as follows: 3 mL of a solution with a concentration of 0.04 mg/mL glycine in 0.025 M borate buffer was placed on the activated with DIC/NHS surface, and the reaction was carried out for 24 h under constant shaking at room temperature. After that, 0.5 mL of glycine solution was withdrawn from the surface of the film and used for the analysis with ninhydrin. Specifically, 2.5 mL of 0.2% ninhydrin solution in ethanol was added to the 0.5 mL of the glycine solution and incubated for 3 h at room temperature. The optical density of the analyzed solutions was measured at 400 nm. The concentration of glycine that did not react with carboxyl groups was determined from the pre-built calibration curve. The latter was built in the range of 0.005 to 0.004 mg/mL under the same conditions as test solutions.

The content of accessible carboxyl groups on the surface (*ν*(*COOH*), mol), expressed in moles, was calculated using the Equation (1):

(1) $$\nu \left(COOH\right)=\frac{{V\times (C}_{0}(Gly)-C_{x}(Gly))}{M(Gly)}$$

where *C*_0_ and *C_x_* are the concentration of glycine in the solution before and after the reaction, respectively, mg/mL; *V* is a sample volume, 3 mL; and *M*(*Gly*) is a molar mass of glycine (75 g/mol).

The grafting density on the surface of the PLA film (*S_COOH_*), expressed in mol/cm^2^, was calculated using the following equation:

(2) $$S_{COOH}=\frac{\nu \left(COOH\right)}{\pi R^{2}}$$

where *R* is the radius of the film equal to 1.5 cm^2^.

##### Amino Groups after Modification with Cys

In order to evaluate the efficiency of the thiol-ene click reaction, the surface of AEMA-modified PLA films was modified with Cys. The amount of bound Cys was determined via the reaction of its amino group with 2,4,6–trinitrobenzene sulfonic acid (TNBS) in solutions before and after modification. It is known that at room temperature, TNBS reacts quantitatively with primary amines to form colored derivatives having a high molar extinction coefficient at 420 nm. The concertation of Cys in test solutions was calculated regarding the calibration curve built for standard Cys solutions in the range of 0.001–0.04 mg/mL in 0.1 M borate buffer, pH 9.4. The analysis was carried out as follows: 0.1 mL of 0.8% TNBS solution in distilled water was added to 3 mL of Cys-containing solution. The resulting solutions were incubated at 30 °C for 30 min, and then the reaction was stopped by adding 1.5 mL of acetate buffer with pH 4.8. The optical density of each solution was measured at 420 nm.

The molar amount of cysteine (*ν*(*Cys*)) immobilized on the surface was determined using the following equation:

(3) $$\nu \left(Cys\right)=\frac{{V\times (C}_{0}(Cys)-C_{x}(Cys))}{M(Cys)}$$

where *C*_0_ and *C_x_* are concentrations of cysteine in the solution before and after the reaction, respectively, mg/mL; *V* is a sample volume equal to 3 mL; and *M*(*Cys*) is a molar mass of cysteine equal to 121 g/mol.

The Cys grafting density (*S_Cys_*) was calculated as the molar amount of Cys on the film surface area:

(4) $$S_{Cys}=\frac{\nu (Cys)}{\pi R^{2}}$$

where *R* is the radius of the film equal to 1.5 cm^2^.

The modification efficacy (*ME*, %) was calculated as follows:

(5) $$ME=\frac{S_{Cys}}{S_{COOH}}\times 100\%$$

where *S_Cys_* and *S_COOH_* are grafting density of cysteine and carboxylic groups, respectively.

#### 2.2.8. Surface Characterization

The detached film was placed on a glass slide, and a 1 µL drop of distilled water was placed on the film surface. In total, about 10 drops were applied to the same film in different zones of the film. Using SCA 20 (version 6.1) software, the drop contour was analyzed, and the wetting angle (*θ*) was measured.

After modification of PLA films with AEMA, the double bonds appearing on the surface of the film were investigated by Raman spectroscopy. The valence vibrations of C=C bonds give an intense band at 1630–1678 cm^−1^. In addition, the analysis of the surface of the AEMA-modified PLA film was carried out by X-ray photoelectron spectroscopy (XPS). The unmodified film served as a comparison sample.

Scanning Electron Microscopy (SEM). The samples were sputter coated with gold–palladium alloy and subsequently viewed at accelerating voltages of 5–15 kV with a Zeiss Supra 55VP (Oberkochen, Germany) scanning electron microscope.

Atomic Force Microscopy (AFM). The films were dried before measurement, and the sheets with an area of 1 cm^2^ were cut out. The scanning area 5 µm × 5 µm was applied. Measurements were carried out with a scanning probe microscope Omicron VT AFM XA 50/500 (CEO Scienta Omicron, Uppsala, Sweden). The free open-source WxSM ver. 5.0 developed 8.2 software (Nanotec, Madrid, Spain) was used for data processing.

Attenuated total reflectance Fourier-transform infrared spectroscopy (ATR-FTIR) was used to analyze the surface of the films using a Shimadzu IRAffinity-1 system equipped with ATR accessory and IRsolution software (version 1.10) (Shimadzu, Tokyo, Japan).

#### 2.2.9. Biological Experiments

Human embryonic kidney cells (HEK 293 cell line) were used in this study. The cells were passaged by changing the nutrient medium every 3 days. The cells were cultivated in DMEM (Dulbecco’s Modified Eagle’s Medium) under humidified conditions using standard protocols. Sterilization of the films involved an irradiation process under a UV lamp (at 365 nm) for 10 min on each side. Before the experiment, films from the unmodified side were glued to the bottom of the wells of a 6-well plate using medical glue BF-6 (Tula Pharmaceutical Factory, Tula, Russia) so that the modified surface was further used as an adhesive surface for cells. Next, 10^5^ cells in 100 μL of medium were placed on the film on the side with the lowest PLys content on the surface (*n* = 3). The plate was placed in the incubator for 2 h, and then 3 mL of culture medium was added to each well. The plate was left in the incubator for 48 h. After completion of the experiment, the medium was removed, and the films were washed 3 times with 0.01 M PBS. Cells on the surface were fixed with 4% formaldehyde solution for 30 min at room temperature. The specimens were washed 3 times with water. The films without staining were analyzed by optical microscopy (CELENA S Digital Imaging System, Logos Biosystems, GE Healthcare, Anyang, Korea). In addition, the films were stained with a calcein–AM solution (4 µg/mL) to visualize the fixed cells. The stained films were examined using fluorescence microscopy (CELENA S Digital Imaging System) at an emission wavelength of 530 nm (an excitation wavelength is 480 nm).

## 3. Results and Discussion

**Figure 2:**
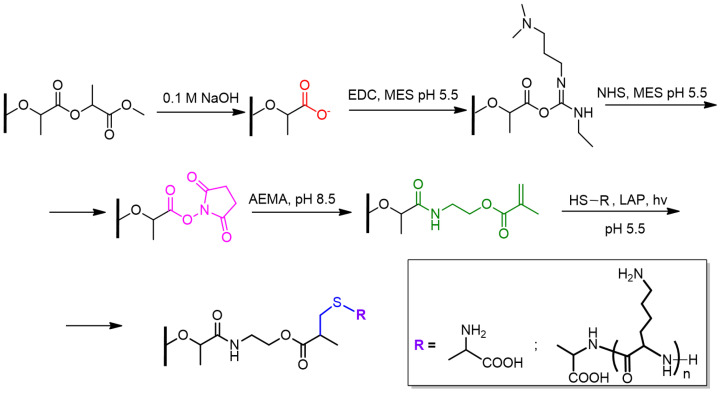
Scheme for modification of PLA-based films. The strategy included surface saponification and activation of generated surface carboxyl groups, modification with AEMA, and further thiol-ene reaction with a thiol-bearing ligand, i.e., Cys or Cys-PLys.

It is known that the efficiency of cell adhesion, migration, and proliferation are affected by the substrate surface properties, including surface charge. Taking this into account, this work was aimed at creating PLA-based films with a surface gradient of cationic polypeptide (PLys) covalently grafted to the PLA surface. A photosensitive thiol-ene click reaction was proposed to provide a gradient one-point covalent attachment of PLys. The idea of the work is that by adjusting the intensity of the light flux, it is possible to control the amount of PLys immobilized on the surface. The scheme of chemical reactions involved in the modification of the surface of PLA-based films is presented in Figure 2. The modification included the generation of carboxylic groups by surface saponification, their further activation, modification with AEMA, and, finally, the thiol-ene reaction with Cys or Cys-PLys.

### 3.1. Synthesis and Characterization of Polymers

#### 3.1.1. PLA

PLA was synthesized by ring-opening polymerization of D,L–lactide in bulk initiated by tin octoate(II), which is recognized by the FDA as a safe and efficient catalyst for this process. The scheme of the reaction can be found in Figure S2 (Supplementary Materials), and the experimental details in Materials and Methods (Section 2.2.1). The structure of the obtained PLA was testified by ^1^H NMR spectroscopy (Figure S3, Supplementary Materials). The characteristics of the synthesized polymer, according to SEC, were the following: *M_n_* = 163,000, *M_w_* = 179,000, and *Đ* = 1.10.

#### 3.1.2. Cys-PLys

**Figure 3:**
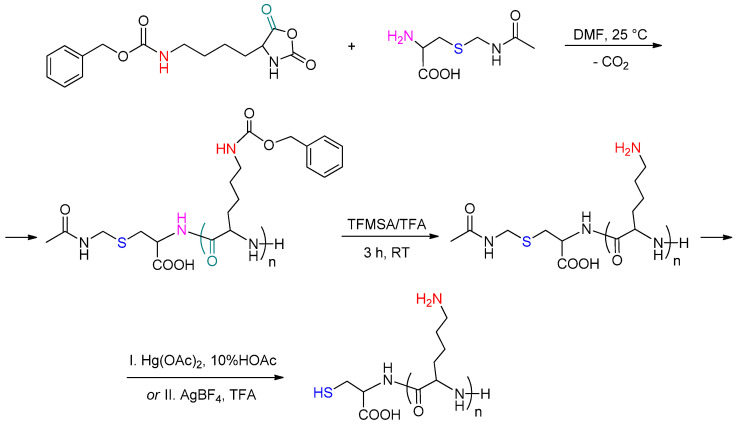
Scheme for the synthesis of Cys-PLys.

In order to accomplish the covalent one-pot attachment of PLys via the thiol-ene reaction, PLys was synthesized so as to have a terminal thiol group (Figure 3). One of the most efficient methods for the synthesis of poly(amino acids) is ring-opening polymerization of the *N*–carboxyanhydride(s) (NCA) of the corresponding amino acid(s). Polymerization of NCA can be initiated by various nucleophiles, the most common of which are primary amines. Use of the latter as initiators, as well as purified anhydrous solvents, allows the synthesis of poly(amino acids) of low dispersity. In this study, Cys(Acm), which has the primary amino group required for ROP NCA as well as a protected thiol group necessary for further thiol-ene reaction, was used as an initiator. Given that NCAs are highly reactive compounds, sensitive to traces of water and unstable during long-term storage, Lys(Z) NCA was synthesized and purified prior to polymerization using a common method based on the use of triphosgen (Figures S4 and S5, Supplementary Materials). Considering that for successful surface modification of PLA-based films, the terminal thiol group in PLys must be sterically accessible to react with the AEMA double bond on the surface, it seemed reasonable to use oligomeric chains of PLys instead of long-chain polymers. With this aim, the sample of Cys(Acm)-PLys(Z) with the following characteristics was synthesized: *M_n_* = 3500, *M_w_* = 4000, and *Đ* = 1.14; the degree of polymerization (*DP*) is 12.

Removal of the Z-protective group from the ε-amino group of Lys was performed with TFMSA/TFA using a known procedure [50]. Elimination of Acm-protection from the thiol group of Cys was realized by two approaches: treatment with mercury(II) acetate [51] and silver tetrafluoroborate (Figure 3). The success of polypeptide deprotection was testified by ^1^H NMR spectroscopy by the disappearance of signals of aromatic and methylene protons of Z-group and methylene and methyl protons of Acm-group (Figures S6 and S7, Supplementary Materials).

In addition, Ellman’s assay was used to estimate the content of thiol groups in the sample after deprotection. The content of free thiol groups in Cys-PLys deprotected with mercury(II) acetate was 79%. At the same time, deprotection with silver tetrafluoroborate resulted in 90% content of thiol groups in the Cys-PLys sample. Based on the results obtained, it can be concluded that deblocking of Acm-protection involving silver tetrafluoroborate was more efficient than deblocking using mercury acetate. Although both methods provide good deprotection efficiency, the second method seems to be more advantageous since traces of Hg(OAc)_2_ may provide potential cytotoxicity. With this in mind, deprotection with silver tetrafluoroborate was used to prepare the Cys-PLys sample for further study.

### 3.2. Fabrication and Modification of PLA-Based Films

#### 3.2.1. Fabrication of PLA-Based Films and Generation of Surface Carboxylic Groups

**Figure 4:**
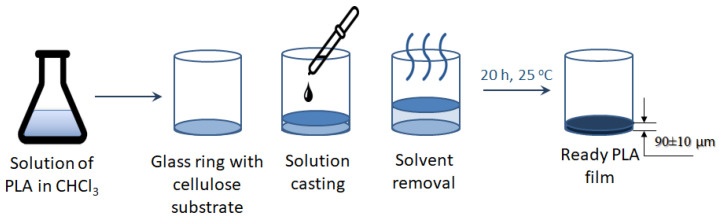
Scheme for the manufacturing of PLA-based films. Light blue color indicates the PLA solution, while dark blue signifies the formed PLA film.

The films were formed by casting the polymer solution onto a cellophane substrate fixed in a glass ring. The thickness of the prepared films was 90 ± 10 µm (Figure 4).

The saponification process degrades the near-surface PLA layer and provides the generation of carboxyl groups. Earlier, it was found that the optimal conditions for surface treatment of films include the use of 0.1 M NaOH solution for 30 min [28]. In this case, there is no significant mass loss of the polymer film. However, with increasing concentration or time of exposure to alkali, degradation of the film is observed. After partial saponification, the contact wetting angle of the PLA-based films decreased, indicating the reduction of the surface hydrophobicity (Table 1).

The content of accessible-for-covalent-modification carboxyl groups on the surface was determined via activation of carboxyl groups (Figure 3) followed by their reaction with small hydrophilic amino acids, i.e., glycine (Gly). The molar quantity of the reactive carboxyl groups is equal to the molar quantity of the attached Gly, whose amount was quantified as a difference of Gly quantity taken for reaction and the quantity of unreacted Gly. It was found that the content of the reactive carboxyl groups per film was (1.0 ± 0.4) × 10^−6^ mol, which corresponds to 1.4 × 10^−7^ mol/cm^2^.

#### 3.2.2. Modification of PLA-Based Films with AEMA

In order to realize the strategy of thiol-ene click reaction on the surface of PLA-based film, it was initially required to introduce a compound containing double bond to the film surface. In our case, AEMA was used as such a compound since it contains the amino-functionality necessary for the reaction with activated surface carboxyl groups (Figure 3), as well as a double bond required for further light-induced click reaction.

**Figure 5:**
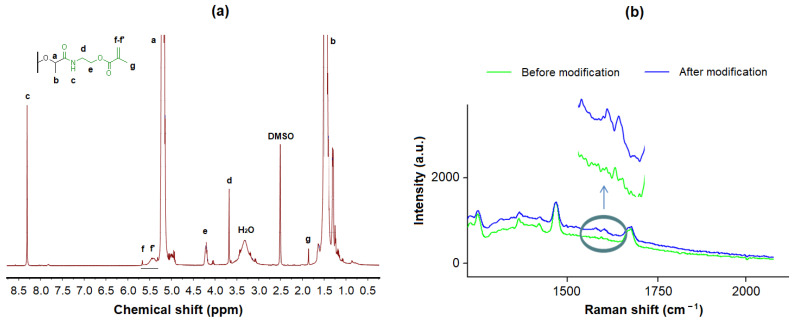
^1^H NMR spectrum (**a**) and fragment of Raman spectrum (**b**) registered for PLA after modification with AEMA. Vertical grey line in the structural formula above ^1^H NMR spectrum (**a**) shows that depicted fragment is attached to the PLA film surface. Horizontal grey line below signals in ^1^H NMR spectrum (**a**) shows that signals of the f,f’ protons in AEMA structure are detected.

First, the modification of PLA with AEMA was confirmed by ^1^H NMR spectroscopy (Figure 5a). The appearance in the PLA spectrum of signals of protons characteristic of methylene, methyl groups, and the AEMA double bond, as well as protons of the amide bond between AEMA and PLA, clearly indicates modification. In addition, the modified PLA was investigated by Raman spectroscopy since double bonds can be clearly distinguished by this kind of spectroscopy. Indeed, the spectrum of PLA modified with AEMA shows the presence of bands corresponding to valence vibrations of the C=C bond at 1650 and 1626 cm^−1^ (Figure 5b). At the same time, no signals were observed in this region in the spectrum of PLA before modification with AEMA.

**Figure 6:**
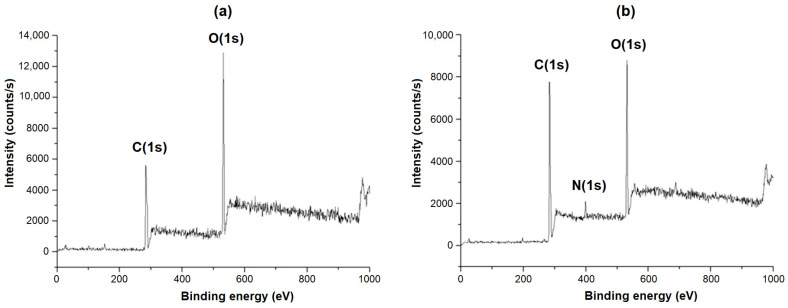
X-ray photoelectron spectra for the PLA before (**a**) and after modification (**b**) with AEMA.

Second, the analysis of the film surface before and after modification with AEMA was carried out by the XPS method (Figure 6, Table 1). The spectrum of PLA-based material after modification with AEMA clearly indicates the appearance of the N(1s) region, which was absent in the PLA before modification. 

The determination of the element percentage in PLA before and after modification also reveals a significant increase in the nitrogen content (Table 2). Thus, it can be concluded that the modification was successful and that PLA-based films contain AEMA on their surface.

In addition, the values of the contact wetting angles were measured for the PLA-based films after modification. The value found after modification with AEMA was 71.1 ± 1.7°, which was close to the value obtained for a partly hydrolyzed surface (Table 1).

**Figure 7:**
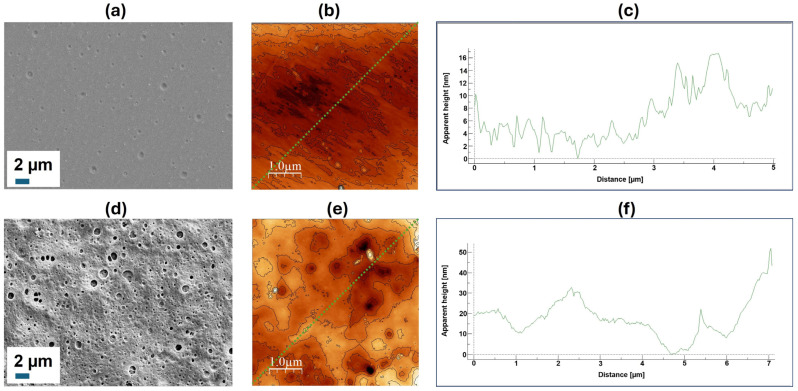
SEM microphotographs (**a**,**d**), AFM microimages (**b**,**e**), and AFM surface profiles (**c**,**f**) of film surfaces before (**a**–**c**) and after (**d**–**f**) modification. SEM microphotographs were obtained at 5k magnification. The green dashed line in images (**b,e)** indicates the corresponding scanning tracks of profiles (**c,f)**.

Unmodified PLA films and those after all stages of modification were analyzed by SEM and AFM (Figure 7). One can observe that initially smooth films become rather rougher and more porous. This is explained by the use of sodium hydroxide solutions to saponify the surface layer.

#### 3.2.3. Thiol-ene Reaction on AEMA-Modified PLA-Based Films

In the first step, cysteine (Cys) was selected as a model compound to examine the light-induced surface thiol-ene reaction of AEMA with the thiol-bearing molecule. The general scheme of modification of PLA-AEMA films is shown in Figure 2.

LAP was used as a photo-sensitive initiator because it successfully decomposes to radicals under the light with a wavelength around 320–420 nm [52]. Thus, the radical thiol-ene click reaction could be induced by visible light. In our case, a 405 nm (25 W) LED source was used for the initiation of the above-described thiol-ene addition reaction. Using Cys as a model compound, the reaction conditions were optimized. The optimal conditions included 10 min exposure time to the light flux and concentrations of 0.5 and 5.0 mg/mL for LAP and Cys, respectively.

**Figure 8:**
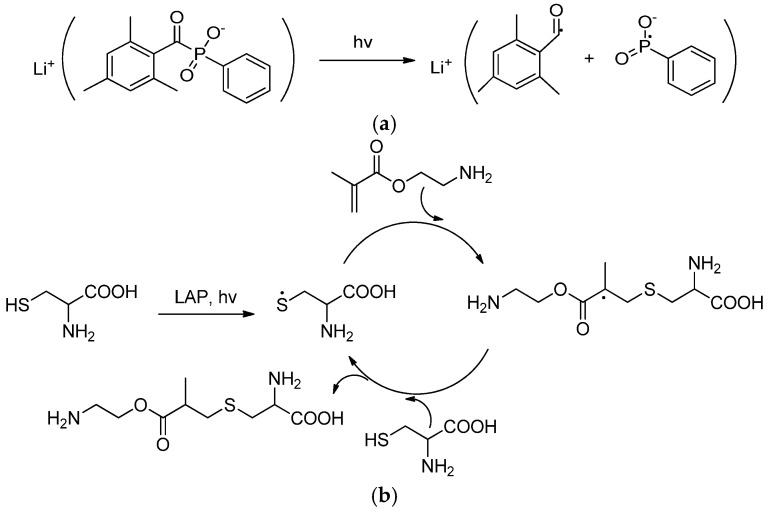
LAP decomposition into radicals upon exposure to light (**a**) and thiol-ene click reaction proceeding by radical mechanism (**b**).

The scheme of the thiol-ene click reaction used in this study is shown in Figure 8. As a result of irradiation, the LAP initiator decomposes into free radicals (Figure 8a). The appeared free radicals detach hydrogen atoms from the thiol of the thiol-containing molecule (Cys) with the formation of thiyl radicals (Figure 8b) [53]. The produced thiyl radicals react with the AEMA double bond to form the intermediate radicals, which, in turn, extract hydrogen atoms from thiols of other Cys molecules to complete the formation of thioether bonds and regenerate thiyl radicals [54].

To quantify the amount of Cys bound due to the thiol-ene reaction, the initial solution of the amino acid and the solution after the binding reaction were analyzed using the TNBS method. The amount of bound cysteine was calculated as a difference in the content of Cys before and after the reaction. The content of immobilized Cys was equal to 1.1 × 10^−7^ mol/cm^2^. The obtained result indicates a sufficiently high grafting density. Earlier, it was shown that grafting 10 fmol/cm^2^ of RGD–peptide to the surface of a glass substrate was sufficient to initiate active adhesion and flattening of fibroblasts on this surface [55]. Thus, it can be expected that the grafting efficacy achieved in this study will be sufficient to exhibit a biological effect.

In addition, regarding the calculated content of carboxyl groups, the modification efficacy (*ME*) is 78.6%, which is a good indicator for the reactions occurring at the interface and inevitably experiencing steric hindrances.

**Figure 9:**
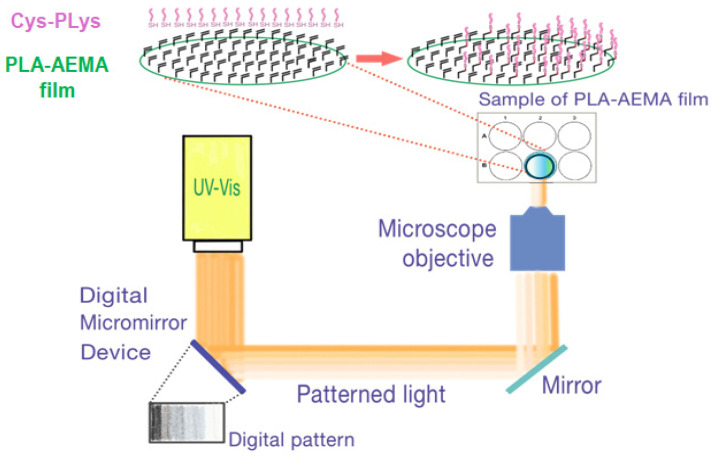
Simplified scheme of the experimental setup used for Cys and Cys-PLys gradient formation on the surface of PLA-AEMA films with application of maskless projection photopatterning method.

The creation of grafted Cys gradients on the surface of PLA-AEMA films was achieved with the use of a maskless projection photopatterning method based on the use of a digital micromirror device, which provides different amounts of light received by the sample film due to the use of digital pattern (Figure 9) [56].

**Figure 10:**
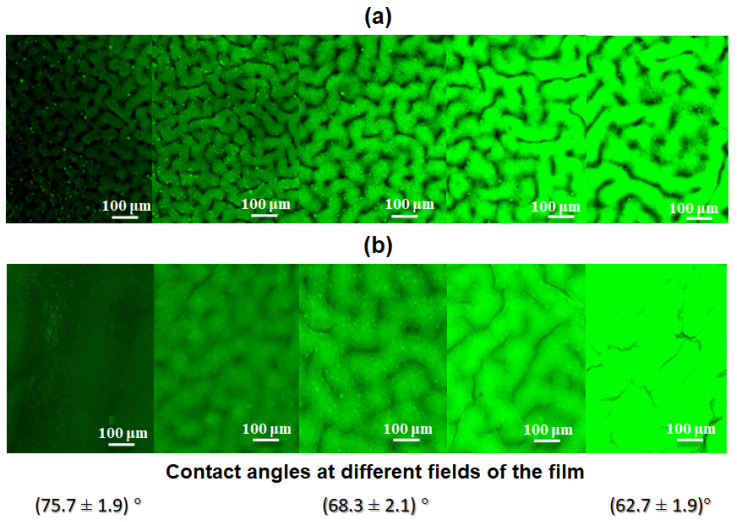
Fluorescence images registered at different parts of the gradient-modified PLA films with Cys (**a**) and Cys-PLys (**b**) via light-induced thiol-ene click reaction and after treatment with Cy3-NHS fluorescent dye (fluorescence microscopy), as well as contact angle values measured at different surface areas of PLA films modified with Cys-PLys (**b**).

In order to visualize the gradient of immobilized Cys on the surface of the film, it was stained with fluorescent dye via covalent modification. Since the grafted Cys contains a free amino group, it can be covalently conjugated with NHS-bearing fluorescent dyes, e.g., Cy3-NHS. For this purpose, a solution of Cy3-NHS in borate buffered solution, pH 8.4, was added to the films. After 2 h, the surface of the films was thoroughly washed with PBS and examined by fluorescence microscopy (Figure 10a). From the presented images, one can observe an increase in fluorescence intensity, indicating an increase in the concentration of bound dye and, consequently, an increase in the number of amino groups proportional to the Cys amount on the surface.

It is known that protein adsorption and cell adhesion are markedly inhibited on a surface grafted with nonionic polymer chains [57,58]. An approach based on surface modification with charged molecules can be used to enhance nonspecific cell adhesion on the surface of materials [59]. However, the highest cell adhesion is usually observed on the surfaces modified with cationic polymers due to the electrostatic interactions with negatively charged phospholipids of the cell membrane [18]. The gradient modification of the PS-grafted PET surface with the cationic polymer, namely poly(*N*–[3–(dimethylamino)propyl]acrylamide methyliodide, significantly enhanced the adhesion of endothelial cells in the region with higher polymer concentration [60].

In this study, the gradient modification of AEMA-functionalized PLA-based films with Cys-PLys was performed using the procedure developed for the Cys grafting via a thiol-ene reaction. Visualization of the obtained gradient-modified films after covalent Cy3-NHS labeling of the bound Cys showed a result similar to the surface modification of AEMA-modified PLA films with Cys (Figure 10). In both cases, increasing the light flux during photoinitiated grafting led to the attachment of a greater quantity of modifying agent (Cys or Cys-PLys). However, in the case of surface immobilization with Cys-PLys, the surface topography was found to be smoothed due to the more elongated chains of the modifying agent.

Measurement of contact angles on the surface of gradient-modified PLA-based films indicated, as expected, surface hydrophilization with increasing grafted PLys content (Figure 10b).

An attempt to evaluate the changes in surface topography using AFM revealed no differences between PLA films treated with alkali solutions and films additionally modified with AEMA and Cys-PLys. The result obtained can be explained by the very pronounced topography changes occurring during the surface hydrolysis of PLA films and the transformation of the initially smooth surface into a rough one. The changes caused by the binding of AEMA, which is a small molecule, and a polypeptide with *DP* = 12 (hydrodynamic radius~1 nm), are not detectable on the rough surface with a height variation of around 10–50 nm (Figure 7f).

**Figure 11:**
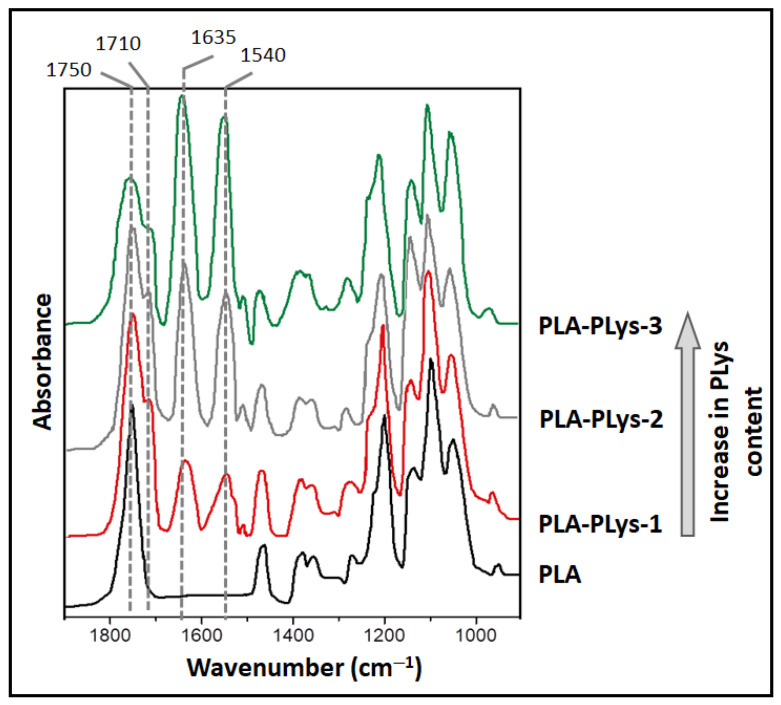
ATR-FTIR spectra of neat PLA film and PLA film gradient-modified with Cys-PLys: PLA-PLys-1 (red)—zone with low grafted PLys content (left side of the film); PLA-PLys-2 (gray) —zone with intermediate grafted PLys content (center of the film); PLA-PLys-3 (green)—zone with high grafted PLys content (right side of the film). The arrow shows the direction of increase in PLys content on the film surface.

A comparative examination of the surface of the neat and gradient-modified PLA films by ATP-FTIR spectroscopy showed that in the spectra of the grafted PLys films, the presence of bands characteristic of the polypeptide can be detected (Figure 11). In all spectra, a specific band at 1750 cm^−1^ corresponding to the valence vibrations of the C=O ester group in PLA was detected. In the spectra of modified films subjected to preliminary hydrolysis of a part of surface carboxyl groups, the appearance of a shoulder at 1710 cm^−1^ corresponding to the valence vibrations of C=O in the carboxyl group was observed. Most importantly, the presence of attached PLys molecules on the PLA surface of modified films was confirmed by the presence of characteristic bands of amide I (1635 cm^−1^) and amide II (1540 cm^−1^), corresponding to the valence vibrations of C=O and N-C=O in the peptide bond of PLys, respectively. Moreover, the spectra recorded at different parts of the gradient-modified film surface (in the center and on the right and left sides) showed a difference in the intensity of amide I and amide II bands, indicating the different amounts of grafted peptide.

Thus, the developed modification approach allowed us to obtain a gradiently functionalized PLA surface.

### 3.3. Biological Examination

The obtained PLys gradient-modified PLA-based films were evaluated for cell adhesion and migration. HEK 293 cells were used in the study as a model cell line. In general, surface coating with polypeptides is a well-known approach for improving cell adhesion [61], including for HEK 293 cells [62]. As for PLys, it is known that it can be cytotoxic in solution, and its cytotoxicity increases with the elongation of the polypeptide chain. Recently, Monnery et al. investigated a set of PLys and poly(ethylene imines) of different molecular weights for their cytotoxicity against A549 (human lung adenocarcinoma) cells [63]. The authors found that cationic polymers with low *DP* have IC_20_ > 1000 μg/mL. Since in our case, PLys was actually an oligomer with *DP* = 12 and was immobilized on the surface but not in solution, cytotoxicity cannot be expected. Moreover, as mentioned above, PLys coatings are widely used in modern cell-culturing technologies to improve cell adhesion. In particular, PLys-coated cell adhesive surfaces are produced both on laboratory [64] and commercial scales [65].

**Figure 12:**
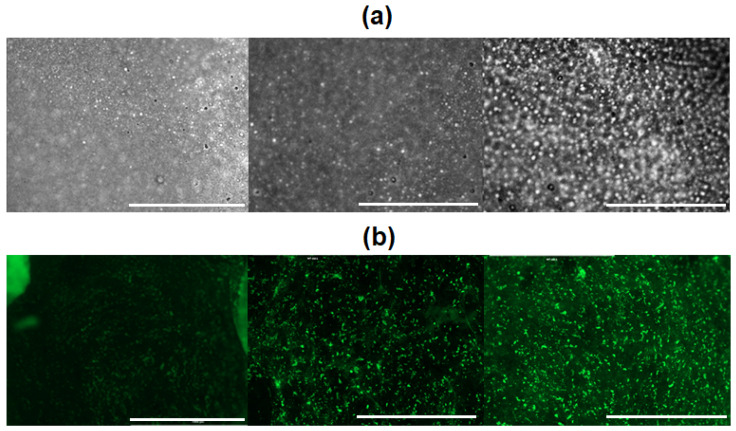
Images of bright-field (**a**) and fluorescence (**b**) microscopy registered at different parts of gradient PLys-grafted PLA-based films with adhered and migrated cells. The concentration of PLys on the surface increases from left to right. Scale bar 1000 µm.

To monitor cell adhesion and migration, cells in 100 µL of the medium were placed on the film on the side with the lowest PLys content on the surface. The plate was closed and incubated for 2 h in a humidified atmosphere. After that, 3 mL of culture medium was added to each well, and the cells were incubated for 48 h. Analysis of the films by optical microscopy revealed a higher cell density on the surface where a higher PLys content was grafted (Figure 12a). The same result was observed by fluorescence microscopy after staining of cells on the surfaces of films with a fluorescent dye (calcein AM) (Figure 12b). The obtained result indicates that during cultivation, cells migrated from the region with a lower content of grafted PLys on the surface to the region with a higher content of grafted PLys.

## 4. Conclusions

An approach for covalent surface modification of PLA-based materials with a cationic polypeptide promoting cell adhesion was developed. The modification strategy involved several steps, such as the generation of PLA surface carboxyl groups, their activation and reaction with an amino group of AEMA, and finally, the photoinduced reaction of an AEMA double bond with the terminal thiol group of PLys. For this purpose, the synthesis of Cys-PLys was carried out, and the presence of a thiol group in the copolymer was confirmed. Each step of modification was testified by a number of physicochemical methods. The developed approach was used to create a PLys gradient on the PLA surface. Using a maskless projection photopatterning method allowed the attachment of the different amounts of PLys to different areas of the PLA film. The latter was confirmed by post-modification with fluorescent dye and visualization by fluorescence microscopy. Finally, selective adhesion and directed migration of HEK 293 cells were detected depending on the PLys content on the surface of PLA material.

In general, the developed approach can be applied for the modification of the surface of other aliphatic polyester-based materials of various design. It should be noted that surface grafting of Cys-PLys can be accomplished by LAP-initiated thiol-ene chemistry in both gradient and non-gradient modes. In the latter case, this method can even be used to covalently modify the surface of PLA/PCL-based micro- and nanoparticles.

## Figures and Tables

**Table 1:** Contact (wetting) angles for neat PLA-based film and PLA-based film after different steps of treatment (H_2_O, 22 °C).

Film	Contact Angle (°)
Neat PLA	91.5 ± 1.4
PLA after treatment with 0.1 M NaOH (30 min)	72.9 ± 1.8
PLA after modification with AEMA	71.1 ± 1.7

**Table 2:** Atom binding energy data and its percentage in PLA before and after modification with AEMA (XPS).

Atom (Orbital)	Binding Energy (eV)	Atom Content (%)	
		Before Modification	After Modification
C(1s)	284.3	63.4	65.7
O(1s)	531.9	33.2	25.2
N(1s)	399.2	0.5	7.0
Si(2p) *	101.4	2.5	2.2

* Since the measurements were performed on a silicon substrate, silicon was also detected.

## Data Availability

The data are available within the article and its Supplementary Materials.

## Supplementary Materials

The following supporting information can be downloaded at https://www.mdpi.com/article/10.3390/polym16202888/s1, Figure S1: Digital pattern, which was prepared in Adobe Photoshop and used for generation of gradient illumination of sample films; Figure S2: Scheme for the synthesis of PLA by ring-opening polymerization of D,L–lactide; Figure S3: ^1^H NMR spectrum of PLA (CDCI_3_, 25 °C); Figure S4: Scheme for the synthesis of Lys(Z) NCA; Figure S5: ^1^H NMR spectrum of Lys(Z) NCA(CDCI_3_, 25 °C); Figure S6: ^1^H NMR spectrum of Cys-PLys(Z) (DMSO-d6, 25 °C); Figure S7: ^1^H NMR spectrum of Cys-PLys (DMSO-d6, 25 °C).
