# Supplementary material for: Gradient Functionalization of Poly(lactic acid)-Based Materials with Polylysine for Spatially Controlled Cell Adhesion
Source: Polymers (Basel). 2024 Oct 14;16(20):2888. doi: 10.3390/polym16202888 (PMC11511340; doi:10.3390/polym16202888)
Supplement: Supplementary file 1 [file polymers-16-02888-s001.zip › polymers-3219420-supplementary.pdf]

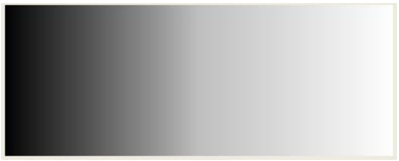

Figure S1. Digital pattern, which was prepared in Adobe Photoshop and used for generation of gradient illumination of sample films.

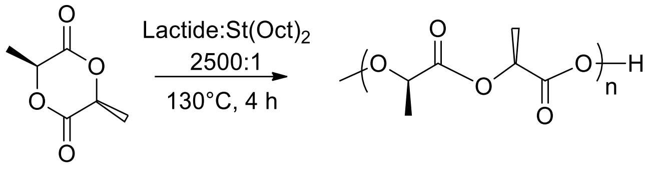

Figure S2. Scheme for the synthesis of PLA by ring-opening polymerization of D,L-lactide.

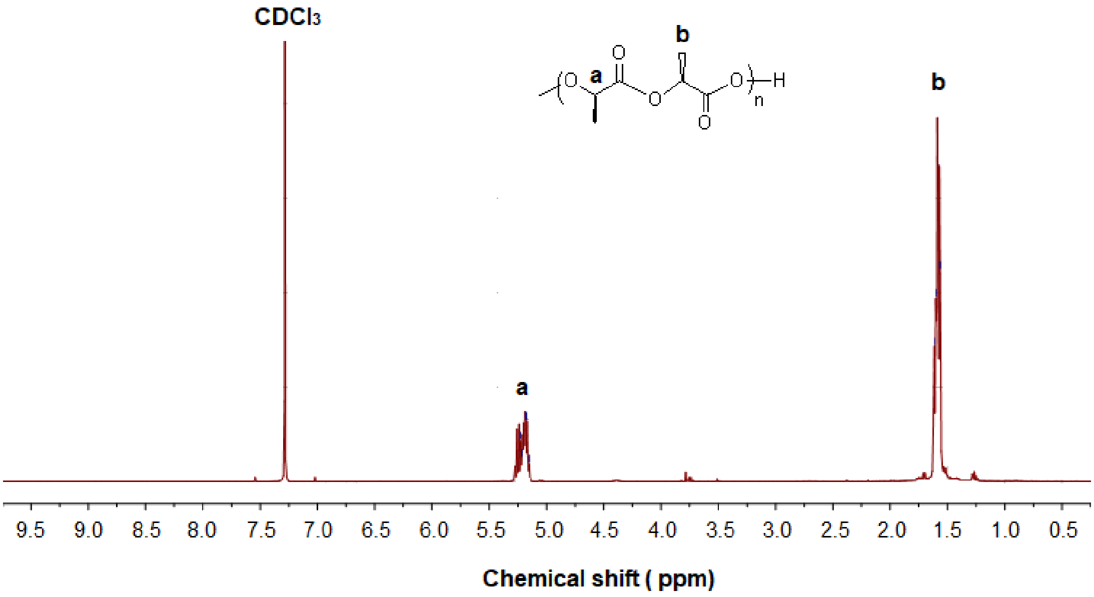

Figure S3. ^1H NMR spectrum of PLA ($CDCl_3$, 25 °C).

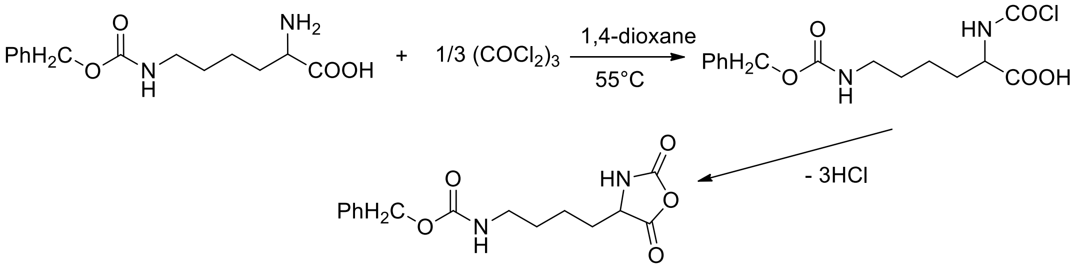

Figure S4. Scheme for the synthesis of Lys(Z) NCA.

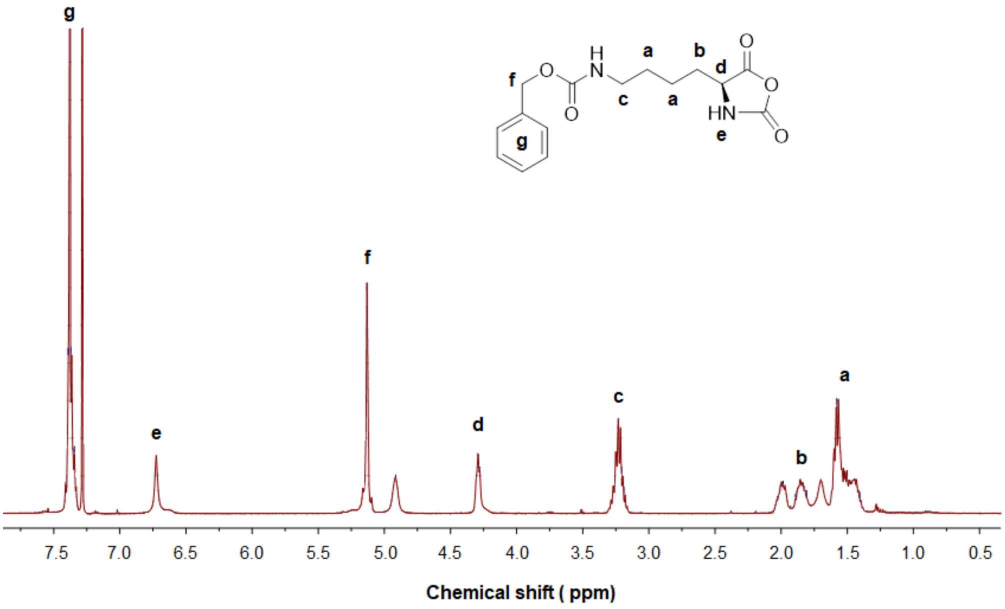

Figure S5. ^{1}H NMR spectrum of Lys(Z) NCA($CDCl_3$, 25 °C).

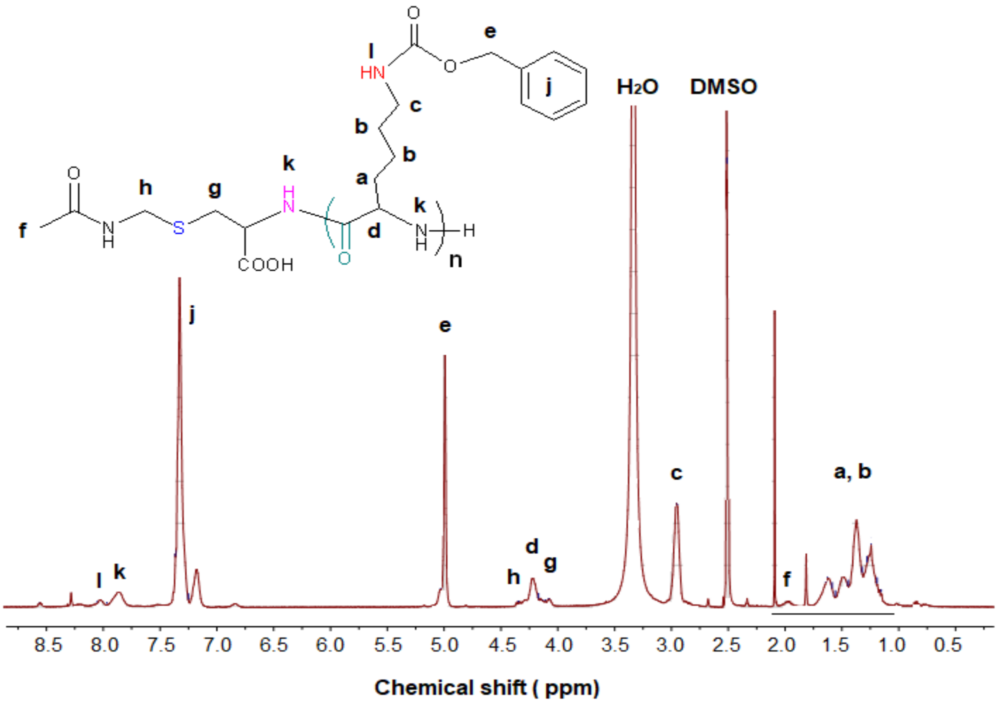

Figure S6. ^{1}H NMR spectrum of Cys-PLys(Z) (DMSO-d6, 25 °C).

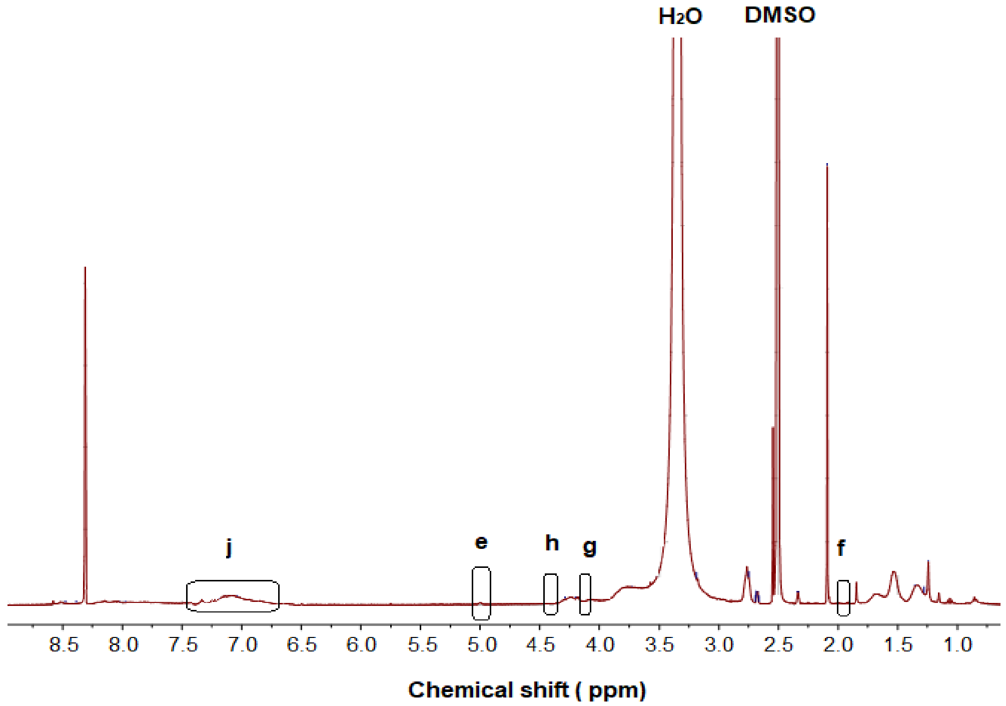

Figure S7. ^{1}H NMR spectrum of Cys-PLys (DMSO-d6, 25 °C). The disappearance of signals of protective ***Z*** (signals ***j*** and ***e*** presented in Figure S5) and ***Acm*** (signals ***f***, ***h*** and ***g*** presented in Figure S5) groups testifies the polypeptide deprotection. Other signals are the same as in Figure S5.
